# *Zanthoxylum bungeanum* root-rot associated shifts in microbiomes of root endosphere, rhizosphere, and soil

**DOI:** 10.7717/peerj.13808

**Published:** 2022-08-04

**Authors:** Li Bin Liao, Xiao Xia Chen, Jun Xiang, Nan Nan Zhang, En Tao Wang, Fu Sun Shi

**Affiliations:** 1 Chengdu Institute of Biology, Chinese Academy of Sciences, Chengdu, China; 2 University of Chinese Academy of Sciences, Beijing, China; 3 CAS Key Laboratory of Mountain Ecological Restoration and Bioresource Utilization & Ecological Restoration and Biodiversity Conservation Key Laboratory of Sichuan Province, Chengdu Institute of Biology, Chinese Academy of Sciences, Chendu, China; 4 Escuela Nacional de Ciencias Biológicas, Instituto Politécnico Nacional, Ciudad de México, México

**Keywords:** *Zanthoxylum bungeanum*, Root-rot diseases, Microbiome, Root endosphere, Rhizosphere

## Abstract

Root-rot disease has lead to serious reduction in yields and jeopardized the survival of the economically and ecologically important *Zanthoxylum bungeanum* trees cultured in Sichuan Province. In order to investigate the interaction between the microbiome and the root-rot disease, a metagenomic analysis was performed to characterize the microbial communities and functions in *Z. bungeanum* root endosphere, rhizosphere and bulk soil with/without root-rot disease. Soil physicochemical properties, microbial population size and enzyme activities were also analyzed for finding their interactions with the root-rot disease. As results, lower total nitrogen (TN) and available phosphorus (AP) contents but higher pH in rhizosphere and bulk soil, as well as lower substrate-induced respiration (SIR) and higher protease activity in bulk soil of diseased trees were found, in comparison with that of healthy trees. Microbial diversity and community composition were changed by root-rot disease in the endosphere, but not in rhizosphere and bulk soils. The endophytic microbiome of diseased trees presented higher Proteobacteria abundance and lower abundances of Bacteroidetes, Firmicutes and dominant fungal phyla. The relative abundances of nitrogen cycle- and carbon cycle-related genes in endophytic microbiomes were different between the diseased and healthy trees. Based on ANOSIM and PCoA, functional profiles (KEGG and CAZy) of microbiomes in rhizosphere and bulk soil shifted significantly between the diseased and healthy trees. In addition, soil pH, TN, AP, SIR, invertase and protease were estimated as the main factors influencing the shifts of taxonomic and functional groups in microbiomes of rhizosphere and bulk soil. Conclusively, the imbalance of root and soil microbial function groups might lead to shifts in the root endosphere-rhizosphere microenvironment, which in turn resulted in *Z. bungeanum* root-rot.

## Introduction

*Zanthoxylum bungeanum* (Rutaceae), with the common name of Chinese pepper or huajiao, is a shrub distributed in tropical and subtropical regions in China (Feng et al., 2017; Li et al., 2020). Traditionally, the fruits of this tree are used as a pungent foodstuff and seasoning, and as herbal medicine for treating toothache and rheumatism in East Asian countries (Zhang et al., 2017a). *Z. bungeanum* also plays an important role for providing ecosystem services in soil and water conservation, barren mountain afforestation, and garden greening (Luo et al., 2020). This tree has been widely cultivated for its high economic and ecological values (Zhuo et al., 2020). However, various plant pathogenic microbes frequently infecting *Z. bungeanum* have caused serious decrease in its yield and quality (Cao et al., 1994).

One of the main diseases of *Z. bungeanum* is root-rot, with symptoms of bursting off the root epidermis, necrosis of the cambium leading to its light-brown discoloration and black spots, and appearance of white flocculent substance on root bark (Qing, Liang & Zhu, 2020). This root disease usually results in trees with smaller leaves and underdeveloped branches, even death of the whole tree (Li et al., 2016b), and it has caused yield loss up to 50% in the main *Z. bungeanum* cultivation regions in China (Zhu & Chen, 1994; Ruan et al., 2022). In our field survey, root-rot was recognized as an important factor to limit *Z. bungeanum* production in the Hanyuan County and Maoxian County of Sichuan Province, the major planting area of this tree in China. *Fusarium solani* has been reported as the main pathogen of *Z. bungeanum* root rot (Li et al., 2006; Tian et al., 2022), while the temperature and wate of soil, as well as the pathogen quantity have been defined as key factors determining the occurrence and severity of this disease (Zhu, Chen & Zeng, 1995). In order to control this disease, several chemical pesticides (such as arasan and metalaxyl mancozeb) are applied to fumigate the soil or the root zone. Based upon the increased concerns about the negative impacts of pesticide application on quality of *Z. bungeanum* fruits and on the ecosystem in recent years, biocontrol of root-rot of *Z. bungeanum* with endosphere and rhizosphere microbes, such as *Bacillus cereus,* has been recommended and tested (Li et al., 2016b). However, there is currently no effective option is available to prevent the root-rot damage for countering the losses.

The root endosphere and rhizosphere are important habitats colonized by diverse and abundant microorganisms, which can promote plant health or growth by mediating nutrient acquisition or producing phytohormones, and by enhancing plant resistance to reverse conditions or phytopathogens; this may also result in soil-borne diseases (Raaijmakers & Mazzola, 2012; Tan et al., 2017a). In general, the relative abundances of major bacterial populations are similar in rhizosphere and in bulk soil, while the microbial community compositions significantly differ between rhizosphere and endosphere of the plants (Trivedi et al., 2020; Zarraonaindia et al., 2015). The interactions between plant roots and microorganisms have an important role to comprehend the functions of microorganisms in the agricultural ecosystems, including protection against the pathogens, host’s nutrient uptake, biodiversity, crop production and food security (Singh et al., 2004; Sessitsch & Mitter, 2015; Narayan et al., 2021). Rhizosphere microbiota could delay flowering by converting tryptophan to the phytohormone indoleacetic acid, which in turn increased and prolonged nitrogen bioavailability (Lu et al., 2018). Under low phosphate conditions, the rhizosphere microorganisms, including arbuscular mycorrhizal fungi and bacterial endophytes, could help the plants to fulfil their phosphate supplement (Bodenhausen et al., 2019). The plant could enrich some microbial groups in rhizosphere by excretion of root exudates, which act as the first line of defense against fungal root pathogens (Mendes et al., 2011). Meanwhile, the endophytic microbiome can provide a second line of defense through selective enrichment of microbial consortia with antimicrobial activities, including production of lytic enzymes, volatiles and antibiotics (Malhadas et al., 2017; Carrión et al., 2019). The successful colonization and subsequent growth of pathogens in plant roots are influenced by the soil microbial community (Wang et al., 2018). Tan et al. (2017b) reported that the root-rot disease affected the community structure and diversity of rhizospheric and root endophytic fungi in continuous cropping of *Panax notoginseng*. Changes in soil environment associated to root-rot disease can play major roles in shaping microbial community composition and activity (Feng et al., 2019).

Although it is well known that *Z. bungeanum* root-rot is caused by *Fusarium solani*, little information has been published about the effects of root-rot disease on soil microbial communities and the root-associated microbiome enrichment process. Previously, long-lasting strong inhibition of the fungal pathogens *Pseudocercospora zanthoxyli* and *Fusarium sambucinum* by eight endophytes of *Z. bungeanum* were reported (Liu, Wang & Song, 2013; Li et al., 2016a). Therefore, investigating the shifts in root-associated microbiome caused by root-rot disease might greatly help us to understand the microbes involved in the development of this disease and the underlying mechanisms of root infection by plant pathogens. Based on the background mentioned above, we investigated the microbial communities associated with roots and in soil of *Z. bungeanum* trees with/without root-rot disease by using the metagenomic methods in the present study. The objects were (1) to illustrate the microbial community and functions in root endosphere, rhizosphere and bulk soil of healthy and diseased trees; (2) to identify the rhizospheric and endophytic potential pathogens relating to root-rot disease; and (3) to determine the relationships among the environmental factors, microbiomes of root compartments, and *Z. bungeanum* diseases for searching the potential hazard control agents.

## Materials and Methods

### Sampling site and strategy

The study site was located in a plantation of *Z. bungeanum* in Maoxian County (103°75′E, 31°77′N) in Sichuan Province, China. Field experiments were approved by Maoxian County May Crisp Agricultural Technology Company (project number: 1011). On the eastern edge of the Tibetan Plateau, this region has a montane temperate climate characterized by mean annual temperature of 9.3 °C, annual precipitation of 850 mm, and 2,237 m of altitude. The soil was classified as Calcic Luvisols according to the IUSS Working Group for World Reference Base for Soil Resources (WRB IWG, 2006). A plantation occupying about 2 ha with 10-year old *Z. bungeanum* trees was selected for this study, where the trees were planted with a distance of 3 m one another and root-rot diseased trees have been infested for more than 5 years according to the local management practices. The average tree height was about 3.0 m.

Five plots (20 m ×15 m) were divided as repeats in the studied area, with a 20 m interval distance between each other. In July 2020, when the diseased *Z. bungeanum* trees exhibited serious root-rot, one root-rot diseased living tree and one healthy tree of *Z. bungeanum* were randomly selected in each plot for sampling the plant roots (for endosphere), rhizosphere soil and bulk soil, which were marked as HE, HR, HB from healthy trees and DE, DR, DB from the diseased trees, respectively. Bulk soil was sampled from the layer of 0–30 cm under the tree canopy where is free from the roots. For root and rhizosphere soil sampling, plant roots were carefully dug out, and transported to laboratory immediately in sterile plastic bags on ice, where the tightly adhering soil particles on roots after gently shaking were brushed off and collected as rhizosphere soil. The brushed roots, the obtained fresh bulk soils, and the rhizosphere soils were immediately stored at 4 °C for the subsequent biological analyses, and parts of them were stored at −20 °C after passing through a two mm sieve for further DNA extraction. An aliquot of each sample was air-dried for the subsequent physicochemical characterization.

### Soil physicochemical properties

After being passed through a 0.15 mm sieve, the air-dried soil samples were used for physicochemical characterization. The total carbon (TC) and TN contents were assayed with a vario MACRO cube CN Elemental analyzer (Elementar Analysensysteme, Langenselbold Germany) (Wang et al., 2016). Soil extracts of NH_4_^+^-N and NO_3_^−^-N in 1 M KCl solution were measured by continuous flow analysis using a SEAL AutoAnalyzer 3 (SealAnalytical, Norderstedt, Germany). AP in soil was estimated by the Olsen method (Olsen & Cole, 1954), and was determined by ICP-OES. The standard method of McLean & Watson (1985) was applied for analyzing the content of available potassium (AK). Soil pH and electrical conductivity (EC) were determined in 1:2.5 and 1:5 (w/w) soil:water suspensions, respectively.

### Microbial population size and activity analyses

For testing the alterations in microbial population size, the chloroform fumigation extraction method (Vance, Brookes & Jenkinson, 1987) was applied for determining the microbial biomass carbon (MBC) content and microbial biomass nitrogen (MBN) content, which were calculated with the formula: (total extractable C (or N) in fumigated soil - total extractable C (or N) in unfumigated soil) ÷ 0.45 (or 0.54); where 0.45 and 0.54 were the efficient factors Kec and Ken for soil C and N extraction, respectively (Vance, Brookes & Jenkinson, 1987). The potentially active biomass was assessed by SIR based on the activity of soil microbial biomass with supplement of glucose as substrate (Lipson, Schmidt & Monson, 1999). Invertase activity was measured according to Xiao et al. (2017), in which the released glucose through the enzyme lysis was quantified from the absorbance at 508 nm after adding 3,5-dini-trosalicylic acid (Ladd & Butler, 1972). Protease activity was also assayed according to Ladd & Butler (1972). Urease activity was tested according to Xiao et al. (2017), in which 5 g of air-dried soil were mixed with one mL of toluene and 10 mL of urea solution (10%, w/v) in 20 mL of citrate buffer (pH 6.7); then the mixture was incubated at 37 °C for 24 h and amino nitrogen was colorimetrically determined at 578 nm. Urease activity was expressed as the amount of NH_3_-N in mg released by 1.0 g of soil during 24 h (Xiao et al., 2017). Cellulase activity was also estimated from the colorimetrical reaction between released glucose and 3,5-dini-trosalicylic acid, and was expressed as amount in mg of released glucose by 1.0 g of soil in 72 h (Guan, Zhang & Zhang, 1986).

### Microbial community analyses

For analysis of endophytes, the roots were washed to eliminate the surface-adherent soil by running tap water, followed by rinsing three times in distilled water. Surface sterilization of root sample was done by immersing 4 min in ethanol (70%, v/v) , 1 min in 2% (w/v) NaClO_3_ solution, and 1 min in 70% ethanol, followed by rinsing three times in sterile distilled water (Tan et al., 2017b; Woźniak et al., 2019). Metagenomic DNA of the endophytic microbiome was extracted with EZNA^®^ Plant DNA Kit (Omega Bio-Tek, Norcross, GA, USA) following the manufacturer’s guidance. Metagenomic DNAs of the rhizosphere and bulk soil samples were extracted separately using the EZNA™ Omega Mag-bind soil DNA kit (Omega Bio-Tek, Norcross, GA, USA), applying the protocol of manufacturer. The concentration, purity and quality of the acquired DNA samples were evaluated with a NanoDrop ND-1000 spectrophotometer (Thermo Fisher Scientific, Waltham, MA, USA) and by electrophoresis in 1% (w/v) agarose gel, respectively.

Metagenome shotgun sequencing libraries were constructed for the DNA samples with insert sizes of 400 bp by using Illumina TruSeq Nano DNA LT Library Preparation Kit. The Illumina HiSeq X-ten platform (Illumina, San Diego, CA, USA) was used for sequencing with PE150 strategy (Personal Biotechnology Co., Ltd. Shanghai, China). The raw sequencing data were screened and filtered to obtain quality-filtered reads as following: (1) trimming the adapter sequences with CutAdapt v1.2.1 (Martin, 2011); (2) trimming the reads with low quality with a sliding-window algorithm; (3) removing the host contamination by aligning the reads to the host genome with BWA (Li & Durbin, 2009). The quality-filtered reads were assembled *de novo* using IDBA-UD (Peng et al., 2012). Coding regions of metagenomic scaffolds with size >300 bp were predicted with MetaGeneMark (Zhu, Lomsadze & Borodovsky, 2010). With CD-HIT, a nonredundant gene catalog was constructed applying 0.95 as the sequence identity cutoff and 0.9 as the minimum coverage cutoff for the shorter sequences. Based on the number of aligned reads, gene abundance of each sample was calculated using soap.coverage (Gu, Fang & Xu, 2013). By aligning them using BLASTN (e value < 0.001) to the NCBI NT database, the lowest common ancestor taxonomy of the non-redundant genes was identified. For the non-redundant genes, DIAMOND (v0.9) was used to obtain their functional profiles by aligning them with the databases of KEGG and CAZy, respectively (Buchfink, Xie & Huson, 2015).

### Statistical analyses

SPSS version 24 was used for calculating the descriptive statistical parameters. Soil physicochemical properties, microbial diversity index, taxonomic and functional composition were compared by ANOVA (one-way analysis of variance), using Duncan’s multiple range test. Pearson correlation coefficients were applied to reveal the correlations between microbial community compositions (phylum level) and soil properties. In order to discover the correlation between compositional/functional variation in the microbiomes, beta diversity measured as Bray–Curtis dissimilarity was estimated in the basis of taxonomic and functional profiles derived from the non-redundant genes ( Bray & Curtis, 1957), and the results were visualized by PCoA (principal coordinate analysis). The relationships between microbiomes and environmental variables were deduced by redundancy analysis (RDA) in R vegan package (Oksanen et al., 2013). The optimal models for microbe–environment relationships were determined by Monte Carlo permutation tests. Correlations among the gene family distances (CAZy and KEGG) in metagenome, the taxonomic compositions in genus level of microbiomes, and the soil characteristics were determined using Mantel test on the Spearman correlation coefficient in R vegan package. The STAMP software was used to test the differences in taxonomic composition of microbiome between the diseased and healthy trees (Parks et al., 2014). All the acquired raw sequences in this study have been submitted to the Sequence Read Archive in NCBI with the accession number SRP312843.

## Results

### Comparison of the soil physicochemical properties

**Figure 1:**
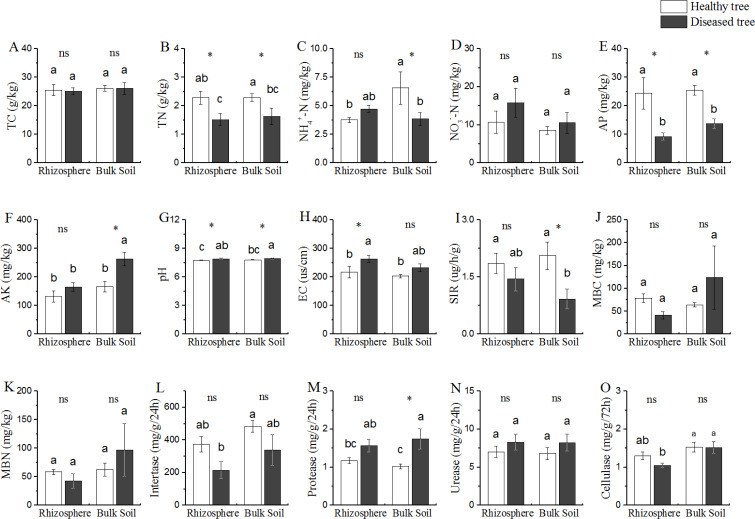
Comparative analysis of (bio-) physicochemical factors rhizosphere and bulk soils. HE, healthy root endosphere; DE, diseased root endosphere; HR, healthy rhizosphere soil; DR, diseased rhizosphere soil; HB, healthy bulk soil; DB, diseased bulk soil; TC, total carbon; TN, total nitrogen; NH${}_{4}^{+}$-N, ammonium nitrogen; NO${}_{3}^{-}$-N, nitrate nitrogen; AP, available phosphorus; AK, available potassium; EC, electrical conductivity; SIR, substrate-induced respiration; MBC, microbial biomass carbon; MBN, microbial biomass nitrogen; ns, not significant. An asterisk (*) indicates *P* < 0.05.

Based upon the comparison between the samples from trees with/without root-rot disease, the soil properties could be divided into three categories (Fig. 1): (1) properties (TC and NO_3_^−^-N contents) presented similar values in all the four soil samples (DR, DB, HR and HB); (2) properties (TN, AP and pH) presented difference between the soils from diseased and healthy trees (DR *vs* HR, and DB *vs* HB), but similar between the two soil samples of diseased trees (DR and DB) and between that of the healthy trees (HR and HB); TN and AP contents were DR = DB < HR = DR (*P* < 0.05), while the cases for pH were reverse (DR = DB > HR = HB); (3) properties (NH_4_^+^-N and AK contents) presented different values in bulk soils of the diseases and healthy trees (DB *vs* HB), but similar in rhizosphere of both trees (DR = HR); NH_4_^+^-N contents were similar in DR, DB and HR, but this value was significantly greater (+ 40% or more) in HB than that in DR, DB and HR; while the AK contents were similar in DR, HR and HB, but it was 59.02% greater in DB than that in DR, HR and HB, respectively.

### Comparison of microbial population size and activity

Microbial population size represented by MBC and MBN, as well as the soil bioactivities of invertase and urease were similar among all the soil samples from trees with/without root-rot disease (DB, DR, HB and HR) (Fig. 1). SIR was lower and protease activity was greater in DB than that in HB samples, while these values were similar in DR and HR. Protease activity in DB was 70.58% greater than that in HB.

### Microbial community composition

**Figure 2:**
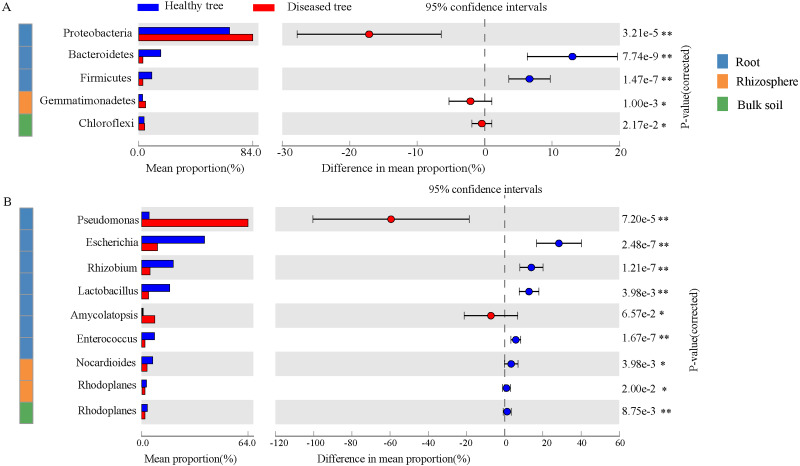
The comparison of the bacterial phyla (A) and the genera (B) with significant differences healthy tree and diseased tree in roots, rhizosphere, and bulk soils. Data are visualized using STAMP analysis with error bars representing Welch’s t-interval.

After quality control, a total of 2,343,370,158 DNA sequences were obtained (Table S1). Significant differences were recorded in taxon numbers at domain level of root endosphere microbiomes between root-rot diseased and healthy trees (DE and HE), with increased bacteria and deceased eukaryota (Table S2). Bulk and rhizosphere microbiomes at domain level were not changed by root-rot disease. Bacteria accounted for 90.69%–99.07% in root and soil microbiomes, followed by Eukaryota. Archaea presented only 0.02%–0.83% of the reads in root and soil microbiomes. The dominant abundant phyla (relative abundance > 1%) include Proteobacteria (35.34%–79.59%), Actinobacteria (10.33%–16.69%), Acidobacteria (0.46%–20.59%), and Bacteroidetes (2.70%–8.23%) (Table S3). Dominance of Proteobacteria was significantly greater in DE samples than that in HE, while Bacteroidetes, Firmicutes and Basidiomycota exhibited an opposite trend (Fig. 2A and Fig. S1). Gemmatimonadetes was more abundant in DR samples than that in HR. The abundances of six most dominant fungal phyla were significantly lower in DE than that in HE samples, and no significant difference was recorded in rhizosphere soils and bulk soils of trees with/without root-rot disease. The abundances of dominant bacterial genera *Pseudomonas* and *Amycolatopsis* were significantly higher in DE than that in HE samples, while the values of *Escherichia*, *Rhizobium* and *Lactobacillus* were reverse. And the abundances of *Nocardioides* and *Rhodoplanes* were significantly less in DR than that in HR samples (Fig. 2B, Table S4). The fungal genera *Rhizophagus*, *Plasmopara*, *Gelatoporia* and *Golovinomyces* presented significantly lower abundances in DE than that in HE samples, while *Pyrenochaeta* and *Fusarium* exhibited an opposite trend. Compared with that in HB, the dominant archaeal genera *Nitrosoarchaeum* and *Nitrosopumilus* were decreased in DB (Fig. S2).

**Figure 3:**
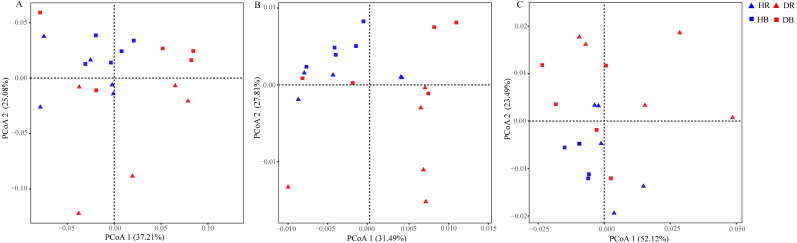
Effects of root-rot on the taxonomic ((A) the genus level) and functional ((B) KEGG pathway level and (C) CAZy level) composition of microbiomes. HR, healthy rhizosphere soil; DR, diseased rhizosphere soil; HB, healthy bulk soil; DB, diseased bulk soil.

The values of alpha-diversity indices Shannon, ACE and Chao were similar across the microbiomes in the four soil samples (HR, DR, HB and DB), and they were significantly greater than the corresponding values in root endosphere (HE and DE). In addition, the microbial community diversity of the microbiome in DE, as estimated by the ACE and Chao indices, was significant higher than that in HE, but their Shannon indices were similar (Table S5). Significant differences between HE and DE samples were detected in ANOSIM (*R* = 0.616, *P* = 0.011) based upon the genus relative abundances (Table S6). The PCoA results of the taxonomic composition of microbiomes demonstrated a difference between HE and DE, while the microbial communities in bulk and rhizosphere soils were not changed by root-rot disease (Fig. 3A and Fig. S3). Compared with HE samples, the presence of 25 phyla covering 736 genera in DE was significantly enhanced or decreased. Compared with healthy tree samples, 5 phyla (597 genera) in DR and 2 phyla (136 genera) in DB varied significantly in response to the root-rot disease.

### Functional shifts in microbial communities

**Figure 4:**
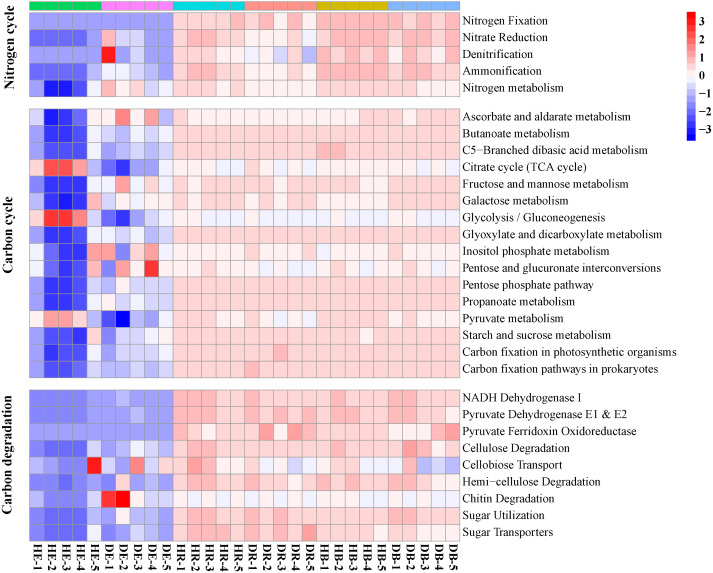
The relative abundances of functional genes responsible for the nitrogen cycle and carbon cycle in the KEGG database. HE, healthy root endosphere; DE, diseased root endosphere; HR, healthy rhizosphere soil; DR, diseased rhizosphere soil; HB, healthy bulk soil; DB, diseased bulk soil.

**Figure 5:**
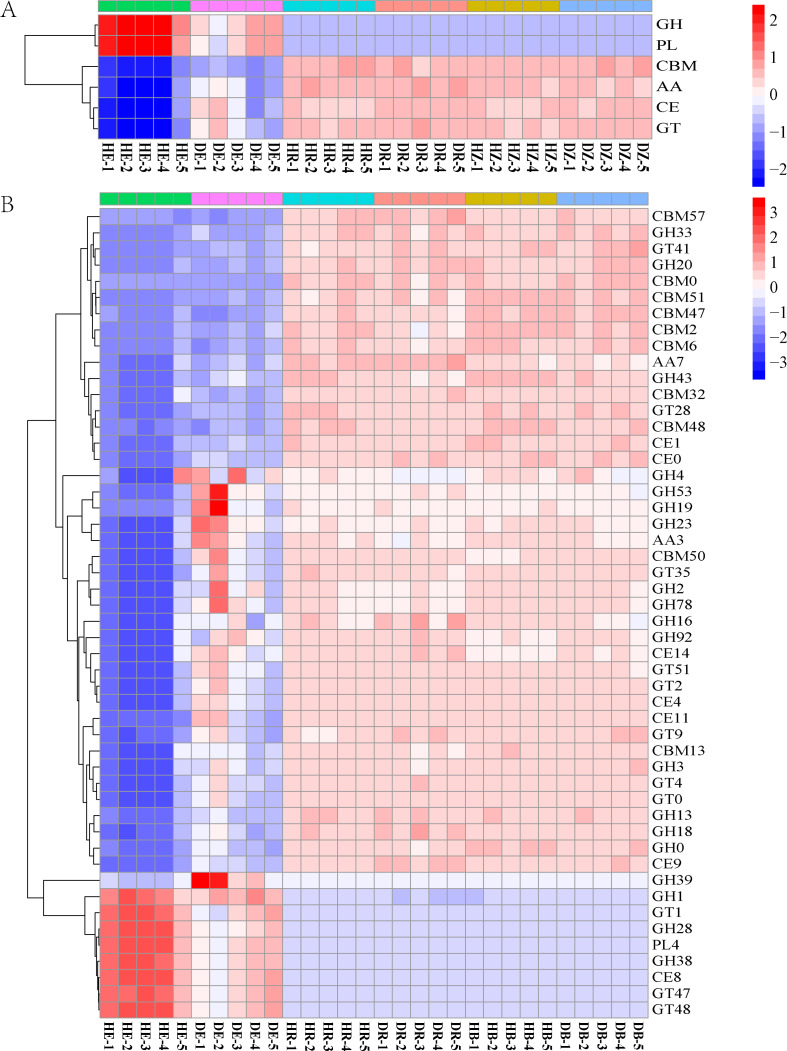
The heatmaps show the distribution of gene classes in the CAZy database (A) and the 50 most abundant CAZy gene families (B). HE, healthy root endosphere; DE, diseased root endosphere; HR, healthy rhizosphere soil; DR, diseased rhizosphere soil; HB, healthy bulk soil; DB, diseased bulk soil.

The relative abundances of functional genes in carbon and nitrogen cycles were compared by annotating the root and soil metagenomic sequences using the KEGG database (Fig. 4, Table S7) and the CAZy database (Fig. 5). In total, 11,566 of KO genes and 336 of CAZy genes were predicted for all samples. PCoA results (Fig. 3 and Fig. S3) showed that root rot disease changed the composition of functional genes in rhizosphere and bulk soils (both KEGG and CAZy databases). The ANOSIM results from KEGG pathway and CAZy level detected significant differences among the microbiomes in root endosphere, rhizosphere and bulk soil between the healthy trees and diseased trees.

The relative abundances of genes related to N and C cycles in DE identified by using the KEGG database were different from that in HE (Fig. 4 and Fig. S4). The relative abundances of genes *nirA* and *napA* related to N cycle decreased in DR and DB, compared with that in HR and HB, respectively (Table S7). The results revealed that 50 dominant CAZy genes in root endosphere were the strong responder of root-rot (Fig. 5); while the dominance of these genes was not varied in bulk and rhizosphere soil between healthy trees and diseased trees.

### Relationship between soil characteristics and the microbiomes

**Figure 6:**
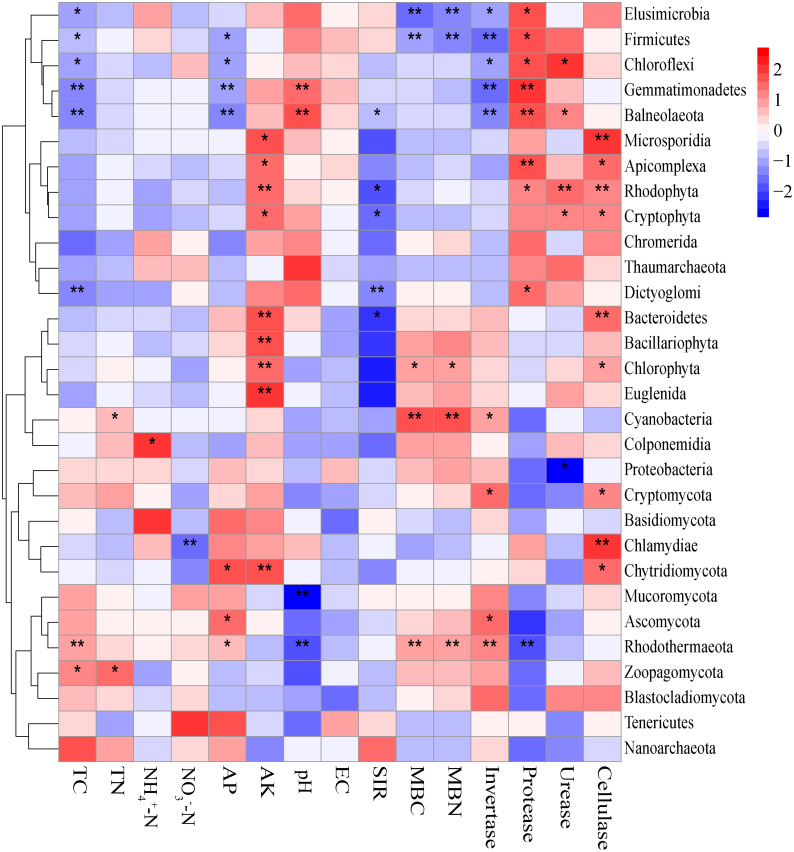
Correlations between significantly changed microbial taxa and environmental factors using the Spearman correlation coefficient (red, positive; blue, negative). An asterisk (*) indicates *P* < 0.05, two asterisks (**) indicate *P* < 0.01.

**Figure 7:**
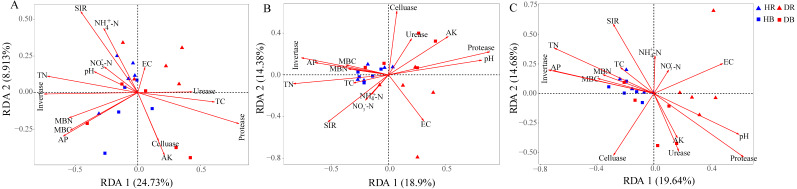
Redundancy analysis (RDA) was used to investigate the relationships between healthy trees and diseased trees with soil properties for the taxonomic ((A) the genus level) and functional ((B) KEGG pathway level and (C) CAZy level) composition of microbiomes. HR, healthy rhizosphere soil; DR, diseased rhizosphere soil; HB, healthy bulk soil; DB, diseased bulk soil. TC, total carbon; TN, total nitrogen, NH${}_{4}^{+}$-N, ammonium nitrogen; NO${}_{3}^{-}$-N, nitrate nitrogen; AK, available potassium; AP, available phosphorus, EC, electrical conductivity; SIR, substrate-induced respiration; MBC, microbial biomass carbon MBN, microbial biomass nitrogen.

The Mantel tests evidenced significant relationships of the soil characteristics with the shifts in the microbial community and the functional composition of microbiomes (*r* = 0. 0.4113 and 0.4234 for microbial community and KEGG genes, respectively) (Table 1). Significant correlations were also found among various soil characteristics and microbial taxa (Fig. 6). Briefly, AK, TC and AP were the most important soil properties that affected the presence of 9, 8 and 7 microbial phyla, respectively. While pH, TN, NO_3_^−^-N, NH_4_^+^-N significantly affected 1 to 4 phyla, and EC had no significant correlation with any of the microbial phyla. The results in Fig. 6 demonstrated that the association between bioactivity of soil and the microbial phyla might reflected the metabolic characters of the microbial groups, such as MBC and MBN presented significantly positive correlation with the phototrophic microorganisms (Cyanobacteria, Rhodothermaeota, Chlorophyta), while the protease and cellulase activities were positively correlated to the protein degraders (Balneolaeota, Gemmatimonadetes) or cellulose degraders (Microsporidia, Chlamydiae). Soil characteristics were closely associated with Balneolaeota and Rhodothermaeota. RDA results showed that soil pH, TN, AP, SIR, invertase and protease were the main factors to regulate the taxonomic and functional compositions in root and soil microbiomes that are associated with *Z. bungeanum*, as revealed by the Monte Carlo significance test (Fig. 7 and Table S8). The microbial functions separated the microbiomes associated with the healthy trees from those of the diseased trees, and the healthy microbiomes were positively correlated with TN, AP, SIR and invertase activity as the mayor factors, as well as TC, MBC and MBN as the minor factors.

**Table 1:** Correlation coefficients estimated by the Spearman method in Mantel test among the soil characteristics. Correlation coefficients estimated by the Spearman method in Mantel test among the soil characteristics (pH, EC, TN, TC, NH_4_^+^-N, NO_3_^−^-N, AK, AP, SIR, MBC, MBN, invertase, protease, urease, cellulase), the UniFrac distances of microbial communities, and the gene family distances (KEGG and CAZy) estimated from metagenome.

	Soil characteristics	Microbial communities	KEGG genes	CAZy genes
Soil characteristics	1	□	□	□
Microbial communities	0.4113^*^	1		
KEGG genes	0.4234^**^	0.7505^**^	1	
CAZy genes	0.2845	0.8876^**^	0.7534^**^	1

**Notes.**

An asterisk (*) indicates *P* < 0.05.

Two asterisks (**) indicate *P* < 0.01.

## Discussion

The root-rot pathogens of *Z. bungeanum* have been well characterized and studied, but the soil characteristics and microbiomes in root endosphere and rhizosphere of the trees with/without root-rot disease have not been clearly described. Our results evidenced lower TN and AP contents, but higher pH in rhizosphere and bulk soils of diseased trees. The higher pH values in soils with diseased plants were also detected previously by Gaitnieks et al. (2016) and Feng et al. (2019), which was attributed to the reduced root exudates or litter input into the soils. So, the higher pH in rhizosphere and bulk soils of diseased plants in our present study might be a result of the decreased root exudates caused by the root-rot development. In a previous study on continuous cropping system with root-rot disease, available N content and pH in soil presented significantly correlation with Sanqi’s survival rate in a Pearson correlation analysis (Xu et al., 2021). Intensive studies on nitrogen nutrient in relation to the host nutrition and disease severity have revealed that TN and nitrogen form are related to the disease severity (Huber & Watson, 1974). Smiley, Collins & Rasmussen (1996) reported lower root-rot levels at low N content for wheat infected primarily by *Fusarium pseudograminearum*. Bean plants showed more severe root-rot in fields supplied with NH_4_-N, since spore germination, a step in the pathogenesis, of *Fusarium solani* was increased by NH_4_-N (Huber & Watson, 1974). In our study, lower TN and NH_4_-N contents were detected in bulk soil of diseased *Z. bungeanum* trees compared with that of healthy trees, which was different from the cases for wheat and bean plants, suggesting that the root-rot disease-inducing environmental factors may vary for different plant species. Also, the lower TN and AP contents but greater AK content in bulk soils around the root-rot diseased *Z. bungeanum* compared with that of the healthy trees might imply the unbalanced nutrients being an inducer (or consequence) for root rot of this plant.

In the present study, metagenomic sequencing was used for estimating the diversity and abundance of the *Z. bungeanum-* associated microbiomes in relation to the root-rot disease, as metagenomic sequencing has been widely applied in related studies (De Azevedo & Quecine, 2017; Carrión et al., 2019). It was reported that root-rot disease of *Panax notoginseng* in continuous cropping changed the internal and external factors of the habitats and the community compositions of the endophytic and rhizospheric microbiomes (Tan et al., 2017b). In our present investigation, clear differences in microbiomes were found between the endophytes in root-rot diseased and healthy *Z. bungeanum* plants, as shown in Fig. 2 and Table S4. Our results demonstrated the increase of bacteria and decease of eukaryote in root endosphere of root-rot diseased plants, with an increase of more than 20 times for *Pseudomonas* and 10 times for *Amycolatopsis*. Among the fungi, abundances decreased for most genera and increased for the known phytopathogens *Phytophthora, Pyrenochaeta* and *Fusarium* (6.8, 3.2, 3.5 times, respectively) in the endosphere of diseased trees. In general, the main root-rot pathogens are fungi, but bacteria and virus also can be pathogens of this kind disease (Bodah, 2017). Previously, *Fusarium sambucinum* as pathogen for *Z. bungeanum* root-rot (Li et al., 2016a), and *Fusarium solani*, *Fusarium oxysporum*, *Phytophthora cactorum, Pseudomonas* sp. as pathogens for *Panax notoginseng* root-rot have been reported (Ma et al., 2013). While the fungal species *Pyrenochaeta terrestris* and *Pyrenochaeta lycopersici* have been verified as pathogen for onion and tomato root-rot (Elsiddig & El Hassan, 2017; Testen et al., 2019). Therefore, the great increase of *Pseudomonas*, *Phytophthora* and *Pyrenochaeta* in root endosphere implied the possibility that they were also the pathogens for *Z. bungeanum* root-rot in the studied field. The increase of *Amycolatopsis* abundance in the diseased roots was interesting, because this actinomycete usually produces antibiotics and is used in biocontrol of bacterial and fungal pathogens. In this case, its increase might refer that it is more competent in the diseased root than other bacteria via its antibiotic character.

In both the natural and agricultural ecosystems, soil microorganisms could profoundly influence plant growth, nutrition and health (Garbeva, Van Veen & Van Elsas, 2004; Shah et al., 2021). For example, the alpha diversity of microbial communities in rhizosphere of diseased *Panax notoginseng* plants was lower than that of its healthy plants (Wu et al., 2015). Apparent changes in soil microbial community composition have been recorded in fields with other root-rot diseased crops, such as *Panax quinquefolius* (Jiang et al., 2019) and *Rehmannia glutinosa* (Wang et al., 2018). However, similar microbial diversities in soils with healthy and diseased *Torreya grandis* plants in subtropical China have been reported (Feng et al., 2019), which was consistent with our results in this study, *e.g.*, no significant differences in microbial diversity and community composition in the soils from healthy and diseased *Z. bungeanum* trees (Fig. 3 and Table S6). For this phenomenon, the fact that key microbial groups or functional species are related to disease control microbial communities, rather than the overall microbial community diversity. In some cases, microbial biomass or diversity was not related to the disease suppression due their functional redundancy; therefore, microbial biomass or diversity could not be used as an effective approach to independently predict soil health (Donegan et al., 1996; Van Bruggen et al., 2006).

Some endophytes possess the ability to control phytopathogens and enhance plant growth, by producing a diverse group of secondary metabolites, extracellular enzymes, antibiotics, or antifungal agents (De Azevedo & Quecine, 2017). To date, however, little information is available about the functional change of endophytic microbiomes in plants infected by root-rot disease. The increased abundances of genes related to the N cycle (nitrate reduction, denitrification and ammonification) and decreased abundances of genes related to the C cycle (cellulose and chitin degradation) in samples of diseased trees compared with that of healthy trees suggested that pathogen infection may promote the nitrogen metabolism and inhibit the activity of some polysaccharide degraders, as reported in other study (Yan et al., 2019). Previously, significantly different potential function patterns, including gene families related to carbohydrate and energy metabolism, were detected between the rhizosphere microbial communities associated with healthy and diseased *Panax notoginseng* plants (Wu et al., 2016). Similarly, functional profiles (KEGG and CAZy) of microbiomes significantly shifted in rhizosphere and bulk soil of diseased trees in comparison with those of healthy trees. Reductions of SIR and increment of protease activity in rhizosphere of diseased trees also support this conclusion. The relative abundances of *nirA* and *napA,* key genes in the assimilatory and dissimilatory nitrate reductions, decreased in DE and DB, respectively, indicating that root-rot disease may slow down soil nitrogen transformation of nitrate to ammonium, decrease amino acid biosynthesis, and enlarge its loss by leaching and denitrification.

Environmental factors, including host plant, soil properties and microclimate, play an important role in shifting the microbial community in rhizosphere and bulk soil (Bossio et al., 1998; Fahey et al., 2020). Soil pH was one of the main abiotic factor to define the growth and phylogenetic diversity of microbes (Fierer & Jackson, 2006; Fahey et al., 2020; Kang et al., 2021). Previous studies have also revealed the significant correlation between soil TN content and microbial gene changes (for both KEGG and CAZy genes) at different successional stages (Zhong et al., 2018). The soil microbes were insensitive to high phosphorus availability in spruce plantation, while soil microbial biomass was increased by phosphorus addition mainly through improving carbon availability and pH (Huang et al., 2016). In our study, soil pH, TN, AP, SIR, invertase and protease were the main factors influencing taxonomic and functional communities of soil microbiomes associated with *Z. bungeanum* (Fig. 7). Although the pH and nutrient contents were different in soils associated with diseased and healthy plants, their microbial diversity index and community composition were similar, subjecting that a high robustness in the microbial community structure to abiotic (such as soil pH, TN) changes was induced by root-rot disease. Furthermore, an intimate relationship was found between the root-rot of *Z. bungeanum* and the imbalance in functional profiles of soil microbiomes in relation to the changes in soil properties. Balneolaeota and Rhodothermaeota as the well-known halophiles have been recorded previously in aquatic and terrestrial habitats (Munoz, Rosselló-Móra & Amann, 2016; Vera-Gargallo & Ventosa, 2018). In the present study, their abundances in rhizosphere were significantly changed by root-rot (Fig. 6), perhaps caused by the hypersaline microenvironment in rhizosphere of root-rot diseased trees.

## Conclusions

This study revealed that root-rot disease is associated with lower TN and AP contents, but greater pH in the rhizosphere and bulk soil. Root-rot disease increased the abundance of phytopathogenic bacteria (*Pseudomonas*) and fungi (*Phytophthora, Pyrenochaeta* and *Fusarium*), and changed the functioning related to metabolic potential and enzyme activities for the nitrogen cycle and carbon cycle in the root endosphere. Microbial functional groups in the rhizosphere and in bulk soils were significantly impacted by plant health status. Soil pH, TN, AP, SIR, invertase and protease were the main factors influencing the taxonomic and functional communities of rhizosphere and bulk soil microbiomes. These results verified that the imbalance of microbial function groups, and enrichment of potential pathogens in root and soil might lead to the root-rhizosphere microenvironment shift from “healthy” to “ill”, and result in *Z. bungeanum* root-rot. Future studies about the impacts of root metabolites on root-rot and root-associated microbiomes may call for a better understanding how the environmental factors influence the *Z. bungeanum* health. Collectively, our results revealed the potential to use the metagenomic analysis as a diagnostic tool in plant disease management, and deepened the understanding of the correlation between soil traits and root-rot disease.

## Supplemental Information

10.7717/peerj.13808/supp-1 Supplemental Information 1 Supplementary figures and tables

10.7717/peerj.13808/supp-2 Supplemental Information 2 Raw data for Fig. 1

10.7717/peerj.13808/supp-3 Supplemental Information 3 Raw data for Table 1

## Additional Information and Declarations

### Field Study Permissions

The following information was supplied relating to field study approvals (i.e., approving body and any reference numbers): Field experiments were approved by Huiyue Yu from the Maoxian County May Crisp Agricultural Technology Company(project number: 1011).

### DNA Deposition

The following information was supplied regarding the deposition of DNA sequences: The data is available at NCBI: SRP312843.

### Data Availability

The following information was supplied regarding data availability: The raw data is available in the Supplementary File.
