# Supplementary material for: Zanthoxylum bungeanum root-rot associated shifts in microbiomes of root endosphere, rhizosphere, and soil
Source: PeerJ. 2022 Aug 4;10:e13808. doi: 10.7717/peerj.13808 (PMC9357373; doi:10.7717/peerj.13808)
Supplement: Supplemental Information 1 [file peerj-10-13808-s001.docx]

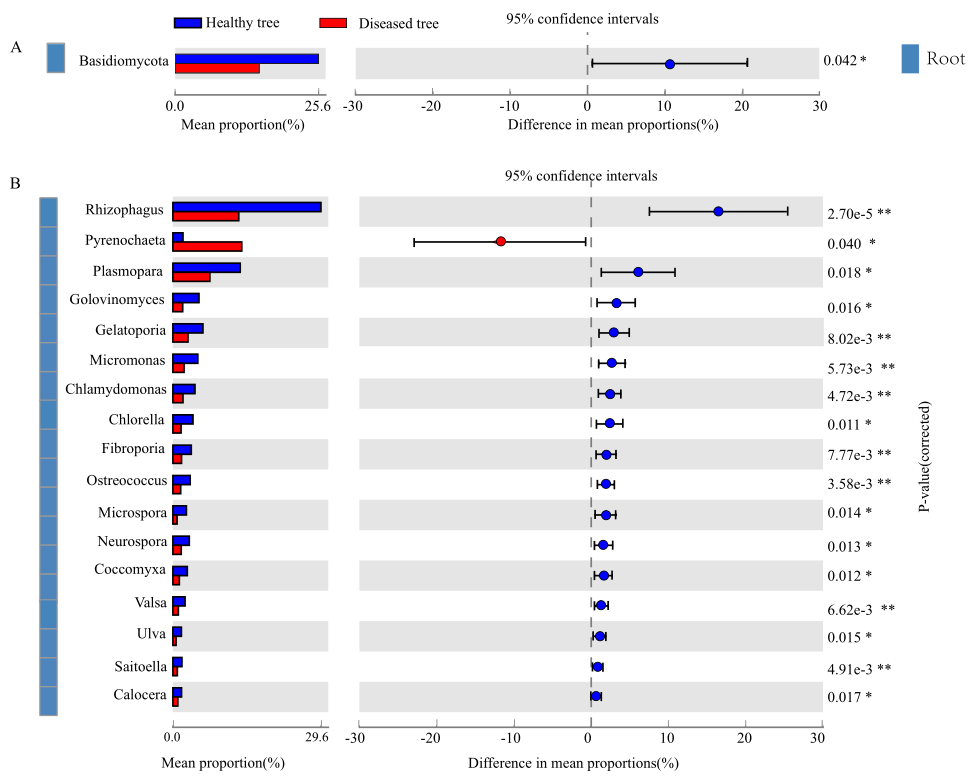


Fig. S1. The comparison of the fungal phyla (A) and the genera (B) with significant differences healthy tree and diseased tree in roots. Data are visualized using STAMP analysis with error bars representing Welch’s t-interval.


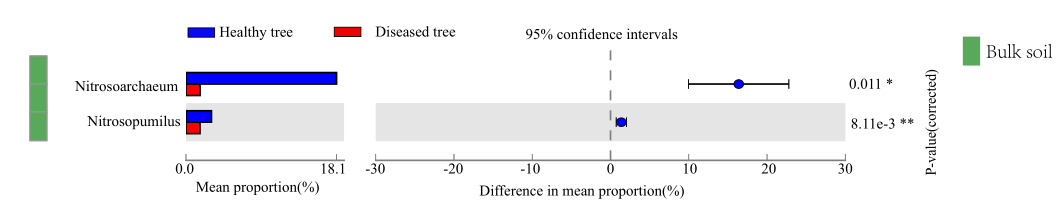


Fig. S2. The comparison of the archaeal genera with significant differences healthy tree and diseased tree in bulk soils. Data are visualized using STAMP analysis with error bars representing Welch’s t-interval.


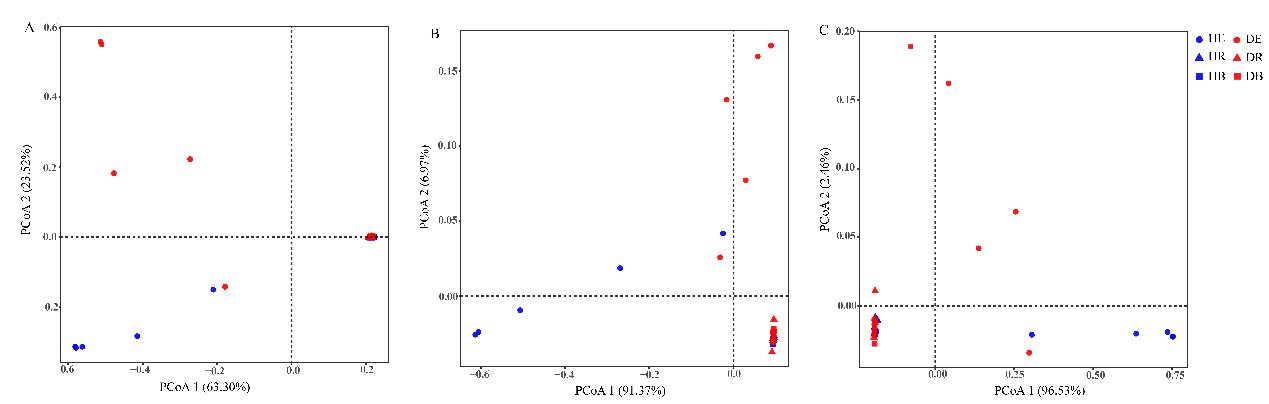


Fig. S3. Effects of root-rot on the taxonomic (a: the genus level) and functional (b: KEGG pathway level and c: CAZy level ) composition of microbiomes. HE: healthy root endosphere, DE: diseased root endosphere, HR: healthy rhizosphere soil, DR: diseased rhizosphere soil, HB: healthy bulk soil, DB: diseased bulk soil.

Fig. S4 Comparative analysis of gene abundance in nitrogen and carbon cycles between healthy tree and diseased tree.

**Supplementary Table S1** Summary of the metagenome sequencing of 30 samples of healthy tree and diseased tree

| Samples | Raw reads | Raw bases (bp) | High quality  Reads (%) | High quality  Data (%) | Contigs | N50 (bp) | N90 (bp) | Min (bp) | Max (bp) |
| --- | --- | --- | --- | --- | --- | --- | --- | --- | --- |
| HE1 | 70282716 | 10542407400 | 99.67 | 99.64 | 872325 | 626 | 352 | 200 | 77949 |
| HE2 | 69308574 | 10396286100 | 99.71 | 99.68 | 795284 | 702 | 364 | 200 | 61689 |
| HE3 | 77071158 | 11560673700 | 99.64 | 99.62 | 819994 | 707 | 366 | 200 | 57943 |
| HE4 | 78729272 | 11809390800 | 99.7 | 99.67 | 846330 | 682 | 363 | 200 | 49212 |
| HE5 | 75820158 | 11373023700 | 99.61 | 99.58 | 1016270 | 594 | 346 | 200 | 64930 |
| DE1 | 77265808 | 11589871200 | 99.63 | 99.61 | 734024 | 585 | 345 | 200 | 633796 |
| DE2 | 68214262 | 10232139300 | 99.51 | 99.49 | 544204 | 513 | 333 | 200 | 649423 |
| DE3 | 77158394 | 11573759100 | 99.58 | 99.56 | 1131126 | 620 | 347 | 200 | 553365 |
| DE4 | 77867792 | 11680168800 | 99.69 | 99.66 | 919113 | 657 | 356 | 200 | 896657 |
| DE5 | 76226216 | 11433932400 | 99.65 | 99.62 | 1044065 | 589 | 344 | 200 | 123787 |
| HR1 | 77204084 | 11580612600 | 99.46 | 99.45 | 1429020 | 426 | 320 | 200 | 145740 |
| HR2 | 72566714 | 10885007100 | 99.47 | 99.46 | 1345463 | 422 | 319 | 200 | 9128 |
| HR3 | 92307564 | 13846134600 | 99.48 | 99.46 | 1729632 | 424 | 320 | 200 | 23794 |
| HR4 | 91276656 | 13691498400 | 99.52 | 99.5 | 2092410 | 441 | 321 | 200 | 8918 |
| HR5 | 83232540 | 12484881000 | 99.47 | 99.46 | 1885520 | 439 | 322 | 200 | 63820 |
| DR1 | 84731960 | 12709794000 | 99.42 | 99.4 | 1538333 | 442 | 319 | 200 | 87834 |
| DR2 | 83205028 | 12480754200 | 99.38 | 99.36 | 2025498 | 473 | 325 | 200 | 34364 |
| DR3 | 76937800 | 11540670000 | 99.47 | 99.46 | 1796203 | 521 | 329 | 200 | 109493 |
| DR4 | 82516910 | 12377536500 | 99.41 | 99.39 | 2025332 | 481 | 326 | 200 | 53559 |
| DR5 | 77084036 | 11562605400 | 99.4 | 99.38 | 1848787 | 491 | 327 | 200 | 51895 |
| HB1 | 76241120 | 11436168000 | 99.44 | 99.42 | 1529440 | 422 | 319 | 200 | 10622 |
| HB2 | 67901536 | 10185230400 | 99.46 | 99.44 | 1213072 | 409 | 318 | 200 | 24940 |
| HB3 | 76482598 | 11472389700 | 99.42 | 99.4 | 1455973 | 416 | 319 | 200 | 66064 |
| HB4 | 72846412 | 10926961800 | 99.44 | 99.42 | 1420299 | 415 | 319 | 200 | 11173 |
| HB5 | 87081878 | 13062281700 | 99.44 | 99.42 | 1773868 | 424 | 320 | 200 | 35718 |
| DB1 | 82419978 | 12362996700 | 99.48 | 99.47 | 1659741 | 425 | 319 | 200 | 40099 |
| DB2 | 70285940 | 10542891000 | 99.49 | 99.47 | 1404131 | 420 | 319 | 200 | 76544 |
| DB3 | 80216390 | 12032458500 | 99.51 | 99.49 | 1805273 | 447 | 322 | 200 | 18292 |
| DB4 | 81424080 | 12213612000 | 99.47 | 99.46 | 1807440 | 453 | 324 | 200 | 79287 |
| DB5 | 79462584 | 11919387600 | 99.54 | 99.52 | 1925070 | 457 | 323 | 200 | 50069 |

HE: healthy root endosphere, DE: diseased root endosphere, HR: healthy rhizosphere soil, DR: diseased rhizosphere soil, HB: healthy bulk soil, DB: diseased bulk soil.

.

**Supplementary Table S2** Comparative analysis of the relative abundance (%) of Archaea, Bacteria, and Eukarya in root and soils of healthy tree and diseased tree

| Domain | HE | DE | HR | DR | HB | DB |
| --- | --- | --- | --- | --- | --- | --- |
| Archaea | 0.02±0.00b | 0.02±0.01b | 0.76±0.02a | 0.83±0.01a | 0.77±0.01a | 0.76±0.05a |
| Bacteria | 93.00±0.99b | 97.95±0.32a | 99.07±0.02a | 99.03±0.08a | 99.02±0.04a | 99.05±0.04a |
| Eukaryota | 6.98±0.99a | 2.03±0.31b | 0.17±0.02c | 0.14±0.01c | 0.21±0.04c | 0.18±0.02c |

HE: healthy root endosphere, DE: diseased root endosphere, HR: healthy rhizosphere soil, DR: diseased rhizosphere soil, HB: healthy bulk soil, DB: diseased bulk soil.

**Supplementary Table S3** Comparative analysis of the domain phyla relative abundance (%) of Archaea, Bacteria, and Eukarya in root and soils of healthy tree and diseased tree

| Domain/phyla | HE | DE | HR | DR | HB | DB |
| --- | --- | --- | --- | --- | --- | --- |
| **Bacteria** |  |  |  |  |  |  |
| Proteobacteria | 56.09±1.1b | 79.59±7.4a | 36.71±0.8c | 35.34±2.2c | 37.31±0.5c | 37.19±0.8c |
| Actinobacteria | 12.17±4.6a | 10.33±4.3a | 16.69±0.9a | 14.27±1.2a | 14.99±0.7a | 13.55±1.6a |
| Acidobacteria | 0.46±0.13b | 0.99±0.62b | 19.26±0.8a | 18.91±1.7a | 20.59±0.4a | 19.91±0.8a |
| Bacteroidetes | 8.23±0.53a | 2.70±0.90b | 2.90±0.23b | 2.73±0.15b | 3.34±0.17b | 3.49±0.16b |
| Firmicutes | 14.07±2.4a | 2.84±0.93b | 0.99±0.02b | 1.04±0.03b | 0.98±0.01b | 0.97±0.03b |
| Chloroflexi | 0.75±0.10c | 0.28±0.12c | 4.29±0.26a | 4.42±0.29a | 3.45±0.07b | 4.16±0.26a |
| Planctomycetes | 0.37±0.16b | 0.43±0.24b | 3.90±0.06a | 3.80±0.12a | 4.02±0.04a | 3.86±0.10a |
| Gemmatimonadetes | 0.04±0.01c | 0.08±0.06c | 2.48±0.12b | 4.59±0.76a | 2.33±0.06b | 3.40±0.59b |
| Verrucomicrobia | 0.19±0.04b | 0.25±0.14b | 2.40±0.14a | 2.54±0.23a | 2.55±0.04a | 2.62±0.17a |
| Nitrospirae | 0.02±0.00c | 0.05±0.04c | 1.15±0.06b | 1.34±0.11ab | 1.29±0.05ab | 1.46±0.14a |
| **Eukaryota** |  |  |  |  |  |  |
| Ascomycota | 1.55±0.33a | 1.04±0.26b | 0.09±0.01c | 0.07±0.01c | 0.12±0.03c | 0.10±0.01c |
| Chlorophyta | 2.47±0.51a | 0.42±0.15b | 0.01±0.00b | 0.01±0.00b | 0.02±0.00b | 0.02±0.01b |
| Mucoromycota | 1.68±0.24a | 0.31±0.10b | 0.02±0.00b | 0.02±0.01b | 0.02±0.00b | 0.02±0.00b |
| Basidiomycota | 0.80±0.13a | 0.17±0.05b | 0.02±0.00b | 0.02±0.00b | 0.02±0.00b | 0.02±0.00b |
| Rhodophyta | 0.12±0.02a | 0.02±0.01b | 0.00±0.00b | 0.00±0.00b | 0.00±0.00b | 0.00±0.00b |
| Chytridiomycota | 0.08±0.02a | 0.02±0.01b | 0.00±0.00b | 0.00±0.00b | 0.00±0.00b | 0.00±0.00b |
| **Archaea** |  |  |  |  |  |  |
| Thaumarchaeota | 0.01±0.00b | 0.01±0.01b | 0.57±0.02a | 0.63±0.06a | 0.58±0.02a | 0.57±0.05a |
| Euryarchaeota | 0.01±0.00c | 0.01±0.01c | 0.15±0.01ab | 0.15±0.01a | 0.14±0.00b | 0.14±0.00ab |

HE: healthy root endosphere, DE: diseased root endosphere, HR: healthy rhizosphere soil, DR: diseased rhizosphere soil, HB: healthy bulk soil, DB: diseased bulk soil.

**Supplementary Table S4** Comparative analysis of the domain genera relative abundance (%) of Archaea, Bacteria, and Eukarya in root and soils of healthy tree and diseased tree

| Domain/genera | HE | DE | HR | DR | HB | DB |
| --- | --- | --- | --- | --- | --- | --- |
| **Bacteria** |  |  |  |  |  |  |
| *Pseudomonas* | 2.08±0.27b | 45.92±15.87a | 1.04±0.05b | 1.20±0.11b | 1.16±0.06b | 1.14±0.15b |
| *Escherichia* | 18.39±3.24a | 4.05±1.34b | 0.04±0.02b | 0.02±0.00b | 0.02±0.01b | 0.02±0.01b |
| *Bradyrhizobium* | 3.80±1.32a | 2.48±0.70a | 4.06±0.18a | 4.00±0.29a | 3.91±0.02a | 3.41±0.23a |
| *Streptomyces* | 3.59±2.73a | 1.59±0.68a | 2.49±0.16a | 2.38±0.14a | 2.08±0.08a | 1.99±0.14a |
| *Sphingomonas* | 0.59±0.30b | 1.05±0.52b | 2.91±0.38a | 3.29±0.79a | 2.76±0.12a | 3.04±0.21a |
| *Rhizobium* | 9.12±1.45a | 2.20±0.72b | 0.55±0.01b | 0.54±0.00b | 0.52±0.01b | 0.58±0.06b |
| *Mesorhizobium* | 1.13±0.19b | 0.84±0.41b | 2.39±0.11a | 2.55±0.22a | 2.03±0.02a | 2.04±0.09a |
| *Solirubrobacter* | 0.19±0.08c | 0.24±0.13c | 2.68±0.08ab | 2.84±0.28a | 2.46±0.11ab | 2.20±0.28b |
| *Lactobacillus* | 8.21±1.47a | 1.77±0.59b | 0.05±0.01b | 0.05±0.00b | 0.05±0.00b | 0.05±0.00b |
| *Nocardioides* | 0.07±0.02c | 0.07±0.04c | 2.79±0.16a | 1.89±0.35b | 2.70±0.15a | 2.43±0.26ab |
| *Nitrospira* | 0.01±0.00c | 0.05±0.05c | 1.90±0.16b | 2.40±0.28ab | 2.15±0.10ab | 2.53±0.31a |
| *Enterobacter* | 2.40±0.42ab | 4.74±0.71a | 0.01±0.00b | 0.01±0.00b | 0.01±0.00b | 0.01±0.00b |
| *Variovorax* | 0.91±0.41a | 1.34±0.71a | 1.16±0.09a | 0.91±0.10a | 1.09±0.04a | 1.07±0.07a |
| *Steroidobacter* | 0.49±0.28a | 1.38±0.70a | 0.93±0.06a | 1.28±0.27a | 0.85±0.03a | 1.01±0.09a |
| *Gaiella* | 0.01±0.00d | 0.01±0.00d | 1.32±0.11ab | 1.62±0.20a | 1.15±0.06bc | 0.99±0.13c |
| *Acidobacterium* | 0.02±0.01c | 0.07±0.06c | 1.18±0.08ab | 1.10±0.13b | 1.33±0.06a | 1.30±0.06ab |
| *Rhodoplanes* | 0.07±0.02d | 0.12±0.06d | 1.20±0.06b | 0.98±0.07c | 1.43±0.05a | 1.18±0.10b |
| *Mycobacterium* | 0.78±0.44ab | 0.26±0.13b | 1.09±0.06a | 0.96±0.04a | 0.99±0.04a | 0.86±0.06a |
| *Lysobacter* | 0.51±0.28bc | 0.18±0.08c | 1.20±0.09a | 0.84±0.07ab | 1.05±0.05a | 1.04±0.16a |
| *Amycolatopsis* | 0.31±0.06b | 3.21±1.63a | 0.33±0.01b | 0.35±0.04b | 0.31±0.02b | 0.30±0.02b |
| **Eukaryota** |  |  |  |  |  |  |
| *Rhizophagus* | 1.77±0.24a | 0.36±0.13b | 0.01±0.00c | 0.02±0.01c | 0.02±0.01c | 0.02±0.01c |
| *Phytophthora* | 0.14±0.03a | 0.95±0.59a | 0.00±0.00a | 0.00±0.00a | 0.00±0.00a | 0.00±0.00a |
| *Plasmopara* | 0.81±0.12a | 0.20±0.07b | 0.00±0.00c | 0.00±0.00c | 0.00±0.00c | 0.01±0.01c |
| *Gelatoporia* | 0.36±0.05a | 0.08±0.03b | 0.00±0.00c | 0.00±0.00c | 0.00±0.00c | 0.00±0.00c |
| *Pyrenochaeta* | 0.09±0.06b | 0.29±0.08a | 0.01±0.00b | 0.01±0.00b | 0.02±0.01b | 0.01±0.00b |
| *Golovinomyces* | 0.33±0.07a | 0.06±0.03b | 0.00±0.00b | 0.00±0.00b | 0.00±0.00b | 0.00±0.00b |
| *Micromonas* | 0.31±0.06a | 0.06±0.02b | 0.00±0.00b | 0.00±0.00b | 0.00±0.00b | 0.01±0.00b |
| *Chlamydomonas* | 0.27±0.05a | 0.06±0.02b | 0.00±0.00b | 0.00±0.00b | 0.00±0.00b | 0.01±0.00b |
| *Fusarium* | 0.06±0.03b | 0.21±0.07a | 0.01±0.00b | 0.01±0.00b | 0.01±0.00b | 0.01±0.00b |
| *Chlorella* | 0.25±0.05a | 0.05±0.02b | 0.00±0.00b | 0.00±0.00b | 0.00±0.00b | 0.01±0.00b |
| *Exophiala* | 0.11±0.05ab | 0.16±0.08a | 0.01±0.00b | 0.01±0.00b | 0.01±0.00b | 0.01±0.00b |
| *Fibroporia* | 0.23±0.04a | 0.05±0.02b | 0.00±0.00b | 0.00±0.00b | 0.00±0.00b | 0.00±0.00b |
| *Ostreococcus* | 0.21±0.04a | 0.04±0.01b | 0.00±0.00b | 0.00±0.00b | 0.00±0.00b | 0.00±0.00b |
| *Neurospora* | 0.20±0.03a | 0.04±0.02b | 0.00±0.00c | 0.00±0.00c | 0.00±0.00c | 0.00±0.00c |
| *Coccomyxa* | 0.18±0.04a | 0.04±0.01b | 0.00±0.00b | 0.00±0.00b | 0.01±0.00b | 0.00±0.00b |
| *Microspora* | 0.17±0.04a | 0.03±0.01b | 0.00±0.00b | 0.00±0.00b | 0.00±0.00b | 0.00±0.00b |
| *Valsa* | 0.15±0.02a | 0.03±0.01b | 0.00±0.00b | 0.00±0.00b | 0.00±0.00b | 0.00±0.00b |
| *Saitoella* | 0.11±0.02a | 0.02±0.01b | 0.00±0.00c | 0.00±0.00c | 0.00±0.00c | 0.00±0.00c |
| *Calocera* | 0.10±0.01a | 0.03±0.01b | 0.00±0.00c | 0.00±0.00c | 0.00±0.00c | 0.00±0.00c |
| *Ulva* | 0.11±0.02a | 0.02±0.01b | 0.00±0.00b | 0.00±0.00b | 0.00±0.00b | 0.00±0.00b |
| **Archaea** |  |  |  |  |  |  |
| *Nitrososphaera* | 0.00±0.00b | 0.00±0.00b | 0.33±0.03a | 0.36±0.09a | 0.30±0.02a | 0.38±0.13a |
| *Nitrosoarchaeum* | 0.00±0.00b | 0.00±0.00b | 0.04±0.01ab | 0.08±0.06a | 0.08±0.01a | 0.01±0.00b |
| *Methanosarcina* | 0.00±0.00b | 0.00±0.00b | 0.03±0.00a | 0.03±0.00a | 0.03±0.00a | 0.03±0.00a |
| *Methanoculleus* | 0.00±0.00b | 0.00±0.00b | 0.01±0.00a | 0.01±0.00a | 0.01±0.00a | 0.01±0.00a |
| *Nitrosopumilus* | 0.00±0.00c | 0.00±0.00 | 0.01±0.00ab | 0.02±0.01a | 0.01±0.00ab | 0.01±0.00bc |
| *Methanothrix* | 0.00±0.00c | 0.00±0.00c | 0.01±0.00ab | 0.01±0.00a | 0.01±0.00b | 0.01±0.00ab |

**Supplementary Table S5** Comparative analysis of the microbial community diversity in root and soils of healthy tree and diseased tree

| Diversity | HE | DE | HR | DR | HB | DB |
| --- | --- | --- | --- | --- | --- | --- |
| Shannon | 6.88±0.63b | 8.03±0.78b | 9.58±0.11a | 9.23±0.14a | 9.55±0.05a | 9.49±0.12a |
| Chao1 | 14286±1862c | 19137±1284b | 28499±86a | 28103±158a | 28377±138a | 28367±79a |
| ACE | 14194±1809c | 18914±1276b | 28181±112a | 27824±137a | 28085±147a | 28079±84a |

HE: healthy root endosphere, DE: diseased root endosphere, HR: healthy rhizosphere soil, DR: diseased rhizosphere soil, HB: healthy bulk soil, DB: diseased bulk soil.

**Supplementary Table S6** ANOSIM analysis results at the genus level for the microbial communities (genus level) and for the KEGG database and the CAZy level.

|  | Genus level | | KEGG pathway | | CAZy level | |
| --- | --- | --- | --- | --- | --- | --- |
|  | R | P | R | P | R | P |
| HE*SE | 0.616 | 0.011 | 0.576 | 0.012 | 0.764 | 0.013 |
| HR*SR | 0.18 | 0.084 | 0.344 | 0.015 | 0.228 | 0.065 |
| HB*SB | 0.244 | 0.086 | 0.492 | 0.01 | 0.292 | 0.032 |

**Supplementary** Table S7 Selected genes related to C and N cycling and their abundances at different successional stages. P values indicate differences among all succession stages.

|  | HE | | DE | | HR | DR | HB | DB | KO_description |
| --- | --- | --- | --- | --- | --- | --- | --- | --- | --- |
| Nitrogen Fixation | | |  | |  |  |  |  |  |
| K02584 | 1.11E-03c | | 1.53E-03c | | 3.74E-02ab | 3.51E-02b | 4.14E-02a | 4.03E-02a | Nif-specific regulatory protein |
| K02585 | 0.00E+00a | | 6.92E-05a | | 0.00E+00a | 0.00E+00a | 7.59E-05a | 1.13E-05a | nitrogen fixation protein NifB |
| K02586 | 0.00E+00b | | 3.02E-05ab | | 1.48E-05ab | 0.00E+00b | 0.00E+00b | 5.61E-05a | nitrogenase molybdenum-iron protein alpha chain |
| K02587 | 0.00E+00a | | 5.20E-05a | | 2.22E-05a | 0.00E+00a | 0.00E+00a | 0.00E+00a | nitrogenase molybdenum-cofactor synthesis protein NifE |
| K02588 | 0.00E+00a | | 7.95E-05a | | 1.43E-05a | 0.00E+00a | 0.00E+00a | 0.00E+00a | nitrogenase iron protein NifH |
| K02589 | 0.00E+00a | | 0.00E+00a | | 0.00E+00a | 5.27E-06a | 0.00E+00a | 0.00E+00a | nitrogen regulatory protein PII 1 |
| K02590 | - | | - | | - | - | - | - | nitrogen regulatory protein PII 2 |
| K02591 | - | | - | | - | - | - | - | nitrogenase molybdenum-iron protein beta chain |
| K02592 | 0.00E+00a | | 7.77E-05a | | 0.00E+00a | 0.00E+00a | 0.00E+00a | 1.82E-05a | nitrogenase molybdenum-iron protein NifN |
| K02593 | - | | - | | - | - | - | - | nitrogen fixation protein NifT |
| K02594 | 6.55E-04a | | 8.04E-04a | | 8.97E-04a | 1.20E-03a | 1.10E-03a | 1.23E-03a | homocitrate synthase NifV |
| K02597 | 0.00E+00a | | 8.20E-05a | | 1.72E-05a | 0.00E+00a | 0.00E+00a | 0.00E+00a | nitrogen fixation protein NifZ |
| K02598 | 1.31E-04a | | 3.74E-04a | | 2.52E-04a | 9.46E-05a | 1.39E-04a | 2.99E-04a | nitrite transporter NirC |
|  |  | |  | |  |  |  |  |  |
| Nitrate Reduction | | |  | |  |  |  |  |  |
| K02567 | 6.10E-05c | | 5.65E-05c | | 1.27E-04ab | 8.83E-05bc | **1.67E-04a** | **1.14E-04b** | nitrate reductase, napA |
| K00368 | 9.96E-06b | | 1.61E-05b | | 1.75E-04a | 1.68E-04a | 1.66E-04a | 1.78E-04a | nitrite reductase (NO-forming) |
| K00369 | - | | - | | - | - | - | - | nitrate reductase-like protein |
| K00370 | 2.42E-05b | | 1.43E-04a | | 1.58E-04a | 1.32E-04a | 1.63E-04a | 1.61E-04a | nitrate reductase 1, alpha subunit |
| K00371 | 1.26E-05b | | 1.18E-04a | | 7.32E-05a | 7.22E-05a | 7.39E-05a | 7.70E-05a | nitrate reductase 1, beta subunit |
| K00372 | 2.59E-04c | | 3.88E-04ab | | 3.83E-04abc | 2.94E-04bc | 4.27E-04a | 4.01E-04ab | nitrate reductase catalytic subunit |
| K00373 | 7.44E-07b | | 9.10E-05a | | 7.37E-06b | 4.04E-06b | 7.23E-06b | 7.15E-06b | nitrate reductase 1, delta subunit |
| K00374 | 2.42E-06b | | 9.61E-05a | | 1.51E-05b | 7.61E-06b | 1.33E-05b | 1.20E-05b | nitrate reductase 1, gamma subunit |
|  |  | |  | |  |  |  |  |  |
| Denitrification | | |  | |  |  |  |  |  |
| K00376 | 3.84E-07b | | 6.83E-05a | | 5.86E-05ab | 5.20E-05ab | 7.87E-05a | 8.15E-05a | nitrous-oxide reductase |
| K07218 | 2.47E-06b | | 6.30E-05a | | 2.76E-05ab | 2.67E-05ab | 3.58E-05ab | 3.56E-05ab | nitrous oxidase accessory protein |
| K04561 | 1.37E-05c | | 8.30E-05ab | | 1.03E-04ab | 5.73E-05bc | 1.26E-04a | 1.03E-04ab | nitric oxide reductase subunit B |
|  |  | |  | |  |  |  |  |  |
| Ammonification | | |  | |  |  |  |  |  |
| K00360 | 1.34E-05ab | | 2.08E-05a | | 6.07E-06ab | 3.17E-06b | 7.10E-06ab | 6.63E-06ab | nitrate reductase (NADH) |
| K00362 | 3.18E-04b | | 5.98E-04a | | 4.24E-04b | 3.65E-04b | 4.44E-04b | 4.52E-04ab | nitrite reductase (NAD(P)H) large subunit |
| K00363 | 6.11E-05b | | 2.08E-04a | | 8.35E-05b | 1.01E-04b | 8.43E-05b | 9.14E-05b | nitrite reductase (NAD(P)H) small subunit |
| K00366 | 6.98E-05b | | 3.18E-05c | | **1.05E-04a** | **6.78E-05b** | 9.38E-05ab | 9.24E-05ab | ferredoxin-nitrite reductase, NirA |
| K00367 | 2.70E-05a | | 1.68E-05a | | 2.27E-05a | 2.50E-05a | 2.92E-05a | 3.34E-05a | ferredoxin-nitrate reductase, NarB |
| K03385 | 2.22E-06c | | 1.86E-05c | | 1.91E-04ab | 1.80E-04b | 2.29E-04a | 2.08E-04ab | cytochrome c-552 |
|  |  | |  | |  |  |  |  |  |
| NADH Dehydrogenase I | | |  | |  |  |  |  |  |
| K00329 | 6.59E-05bc | | 6.54E-05c | | 1.02E-04ab | 1.22E-04a | 1.11E-04a | 1.09E-04a | NADH dehydrogenase |
| K00330 | 1.54E-04c | | 2.85E-04b | | 4.50E-04a | 4.59E-04a | 4.13E-04a | 4.27E-04a | NADH-quinone oxidoreductase subunit A |
| K00331 | 1.36E-04c | | 2.53E-04b | | 5.58E-04a | 6.26E-04a | 5.66E-04a | 5.77E-04a | NADH-quinone oxidoreductase subunit B |
| K00332 | 1.31E-04b | | 1.15E-04b | | 3.97E-04a | 4.27E-04a | 3.91E-04a | 4.12E-04a | NADH-quinone oxidoreductase subunit C |
| K00333 | 2.89E-04b | | 2.30E-04b | | 1.08E-03a | 1.11E-03a | 1.12E-03a | 1.08E-03a | NADH-quinone oxidoreductase subunit D |
| K00334 | 1.28E-04c | | 2.34E-04b | | 2.79E-04ab | 3.05E-04a | 2.90E-04ab | 2.93E-04ab | NADH-quinone oxidoreductase subunit E |
| K00335 | 2.78E-04c | | 4.19E-04b | | 1.01E-03a | 1.02E-03a | 1.00E-03a | 1.00E-03a | NADH-quinone oxidoreductase subunit F |
| K00336 | 2.88E-04c | | 4.77E-04b | | 8.54E-04a | 8.62E-04a | 8.76E-04a | 8.66E-04a | NADH-quinone oxidoreductase subunit G |
| K00337 | 2.03E-04c | | 3.54E-04b | | 9.22E-04a | 9.15E-04a | 9.22E-04a | 9.21E-04a | NADH-quinone oxidoreductase subunit H |
| K00338 | 1.22E-04d | | 2.74E-04c | | 5.21E-04b | 5.95E-04a | 5.13E-04b | 5.17E-04b | NADH-quinone oxidoreductase subunit I |
| K00339 | 1.16E-04c | | 2.48E-04b | | 4.09E-04a | 4.43E-04a | 4.27E-04a | 4.13E-04a | NADH-quinone oxidoreductase subunit J |
| K00340 | 9.74E-05d | | 1.64E-04c | | 3.21E-04ab | 3.48E-04a | 3.13E-04b | 3.31E-04ab | NADH-quinone oxidoreductase subunit K |
| K00341 | 3.25E-04b | | 4.91E-04b | | 1.46E-03a | 1.41E-03a | 1.46E-03a | 1.43E-03a | NADH-quinone oxidoreductase subunit L |
| K00342 | 2.40E-04c | | 4.11E-04b | | 1.21E-03a | 1.21E-03a | 1.24E-03a | 1.21E-03a | NADH-quinone oxidoreductase subunit M |
| K00343 | 2.33E-04c | | 3.95E-04b | | 1.04E-03a | 9.63E-04a | 9.95E-04a | 9.99E-04a | NADH-quinone oxidoreductase subunit N |
|  |  | |  | |  |  |  |  |  |
|  | |  | | Pyruvate Dehydrogenase E1 & E2 | | |  |  |  |
| K00156 | 1.25E-04a | | 1.32E-04a | | 9.72E-05a | 1.01E-04a | 9.50E-05a | 9.88E-05a | pyruvate dehydrogenase (quinone) |
| K00158 | 0.00E+00d | | 8.40E-08d | | 4.46E-05b | 4.77E-05ab | 3.44E-05c | 5.19E-05a | pyruvate oxidase |
| K00161 | 3.74E-04b | | 3.84E-04b | | 1.00E-03a | 1.04E-03a | 9.30E-04a | 9.20E-04a | pyruvate dehydrogenase E1 component subunit alpha |
| K00162 | 3.10E-04b | | 3.17E-04b | | 8.76E-04a | 9.05E-04a | 8.53E-04a | 8.10E-04a | pyruvate dehydrogenase E1 component subunit beta |
| K00163 | 4.16E-04b | | 5.93E-04b | | 1.26E-03a | 1.20E-03a | 1.34E-03a | 1.25E-03a | pyruvate dehydrogenase E1 component |
| K00627 | 2.68E+00a | | 4.52E-01b | | 9.20E-02b | 8.77E-02b | 9.23E-02b | 9.15E-02b | pyruvate dehydrogenase E2 component (dihydrolipoamide acetyltransferase) |
| K00898 | 1.53E-05a | | 9.94E-06a | | 1.18E-06b | 6.04E-07b | 7.18E-07b | 3.68E-07b | pyruvate dehydrogenase kinase |
|  |  | |  | |  |  |  |  |  |
|  | |  | | Pyruvate Ferridoxin Oxidoreductase | | |  |  |  |
| K00169 | 0.00E+00b | | 0.00E+00b | | 7.66E-05a | 8.45E-05a | 8.59E-05a | 8.19E-05a | pyruvate ferredoxin oxidoreductase, alpha subunit |
| K00170 | 0.00E+00b | | 0.00E+00b | | 7.17E-05a | 8.89E-05a | 7.61E-05a | 7.96E-05a | pyruvate ferredoxin oxidoreductase, beta subunit |
| K00171 | 0.00E+00c | | 0.00E+00c | | 4.17E-05b | 5.58E-05a | 3.95E-05b | 3.85E-05b | pyruvate ferredoxin oxidoreductase, delta subunit |
| K00172 | 0.00E+00b | | 0.00E+00b | | 1.76E-05a | 1.55E-05a | 1.76E-05a | 1.77E-05a | pyruvate ferredoxin oxidoreductase, gamma subunit |
|  |  | |  | |  |  |  |  |  |
| Cellulose Degradation | | |  | |  |  |  |  |  |
| K05349 | 2.09E-03a | | 1.04E-03b | | 8.00E-04b | 7.36E-04b | 8.17E-04b | 8.31E-04b | beta-glucosidase |
| K05350 | 2.81E-04a | | 2.31E-04a | | 3.53E-04a | 3.19E-04a | 3.17E-04 | 3.27E-04a | beta-glucosidase |
| K01187 | 3.78E-04b | | 3.70E-04b | | 5.93E-04a | 5.78E-04a | 5.42E-04ab | 5.92E-04a | alpha-glucosidase |
| K01188 | 1.55E-03a | | 2.10E-04b | | 5.38E-07b | 2.46E-07b | 5.98E-07b | 5.16E-07b | beta-glucosidase |
| K01222 | 6.20E-05a | | 4.77E-05a | | 8.23E-05a | 6.95E-05a | 6.85E-05a | 6.76E-05a | 6-phospho-beta-glucosidase |
| K01223 | 0.00E+00b | | 1.90E-04a | | 2.86E-06b | 5.47E-06b | 2.34E-06b | 4.45E-06b | 6-phospho-beta-glucosidase |
| K01179 | 4.00E-04a | | 3.45E-04a | | 2.97E-04a | 3.09E-04a | 2.95E-04a | 3.28E-04a | endoglucanase |
| K00702 | 0.00E+00c | | 3.19E-06c | | 2.71E-05a | 2.19E-05ab | 2.08E-05ab | 1.90E-05b | cellobiose phosphorylase |
|  |  | |  | |  |  |  |  |  |
| Cellobiose Transport | | |  | |  |  |  |  |  |
| K10240 | 7.58E-05a | | 3.88E-05ab | | 2.65E-05b | 1.82E-05b | 2.53E-05b | 1.75E-05b | cellobiose transport system substrate-binding protein |
| K10241 | 4.87E-05a | | 3.45E-05ab | | 2.57E-05ab | 1.90E-05ab | 1.61E-05ab | 1.16E-05b | cellobiose transport system permease protein |
| K10242 | 1.03E-05a | | 1.73E-05a | | 0.00E+00a | 0.00E+00a | 0.00E+00a | 0.00E+00a | cellobiose transport system permease protein |
| K01225 | 3.46E-05a | | 3.03E-05ab | | 1.86E-05b | 1.25E-05b | 1.46E-05b | 1.34E-05b | cellulose 1,4-beta-cellobiosidase |
|  |  | |  | |  |  |  |  |  |
|  | |  | | Hemi-cellulose Degradation | | |  |  |  |
| K01218 | 2.94E-05a | | 4.48E-05a | | 3.89E-05a | 2.23E-05a | 3.39E-05a | 3.82E-05a | mannan endo-1,4-beta-mannosidase |
| K01805 | 9.18E-05c | | 1.99E-04b | | 3.22E-04a | 3.07E-04a | 3.27E-04a | 3.09E-04a | xylose isomerase |
|  |  | |  | |  |  |  |  |  |
| Chitin Degradation | | |  | |  |  |  |  |  |
| K03791 | 4.62E-05b | | 2.25E-04a | | 3.18E-05b | 3.20E-05b | 3.31E-05b | 2.74E-05b | putative chitinase |
| K01183 | 5.79E-04b | | 2.37E-04a | | 1.33E-04b | 1.33E-04b | 1.41E-04b | 1.40E-04b | chitinase |
| K01452 | 7.20E-07ab | | 0.00E+00b | | 1.38E-06a | 1.52E-06a | 1.47E-06a | 1.22E-06ab | chitin deacetylase |
|  |  | |  | |  |  |  |  |  |
| Sugar Utilization | | |  | |  |  |  |  |  |
| K01804 | 3.48E-05b | | 7.87E-05a | | 9.67E-05a | 7.31E-05ab | 7.81E-05a | 7.32E-05ab | L-arabinose isomerase |
| K01805 | 9.18E-05c | | 1.99E-04b | | 3.22E-04a | 3.07E-04a | 3.27E-04a | 3.09E-04a | xylose isomerase |
| K00845 | 1.86E-04c | | 3.95E-04b | | 5.89E-04a | 6.63E-04a | 5.45E-04a | 6.19E-04a | glucokinase |
| K00847 | 2.90E-04a | | 3.23E-04a | | 1.99E-04b | 1.98E-04b | 1.95E-04b | 1.95E-04b | fructokinase |
| K00849 | 4.51E-05c | | 7.15E-05c | | 1.82E-04b | 1.88E-04ab | 1.75E-04b | 2.18E-04a | galactokinase |
| K00886 | 7.39E-05a | | 6.64E-05a | | 1.02E-04a | 9.15E-05a | 1.09E-04a | 1.14E-04a | polyphosphate glucokinase |
| K00854 | 2.01E-04b | | 3.31E-04ab | | 3.59E-04a | 3.57E-04a | 3.25E-04ab | 3.69E-04a | xylulokinase |
| K12308 | 1.86E-04a | | 1.63E-04a | | 1.09E-04a | 8.43E-05a | 7.37E-05a | 1.04E-04a | beta-galactosidase |
| K02793 | 2.24E-05c | | 6.77E-05b | | 1.14E-04a | 1.01E-04a | 1.20E-04a | 1.06E-04a | PTS system, mannose-specific IIA component |
|  |  | |  | |  |  |  |  |  |
| Sugar Transporters | | |  | |  |  |  |  |  |
| K10188 | 2.60E-05a | | 1.62E-05a | | 1.17E-05a | 9.46E-06a | 6.80E-06a | 1.02E-05a | lactose/L-arabinose transport system substrate-binding protein |
| K10189 | 7.65E-06a | | 1.69E-05a | | 8.43E-06a | 7.45E-06a | 6.49E-06a | 6.53E-06a | lactose/L-arabinose transport system permease protein |
| K10190 | 8.08E-06a | | 1.33E-05a | | 3.84E-06a | 4.89E-06a | 4.83E-06a | 3.98E-06a | lactose/L-arabinose transport system permease protein |
| K10191 | 1.93E-05a | | 1.48E-05a | | 7.27E-06a | 6.25E-06a | 4.43E-06a | 7.45E-06a | lactose/L-arabinose transport system ATP-binding protein |
| K10546 | 8.99E-05a | | 7.21E-05a | | 4.67E-05a | 2.67E-05a | 3.38E-05a | 3.03E-05a | putative multiple sugar transport system substrate-binding protein |
| K10547 | 7.49E-05a | | 6.18E-05ab | | 4.00E-05ab | 2.44E-05b | 3.18E-05b | 3.30E-05ab | putative multiple sugar transport system permease protein |
| K10548 | 9.84E-05a | | 6.93E-05ab | | 4.81E-05ab | 3.60E-05b | 5.13E-05ab | 4.57E-05ab | putative multiple sugar transport system ATP-binding protein |
| K02025 | 1.10E-03a | | 7.49E-04a | | 1.25E-03a | 1.28E-03a | 1.10E-03a | 1.08E-03a | multiple sugar transport system permease protein |
| K02026 | 1.03E-03a | | 7.48E-04a | | 1.25E-03a | 1.32E-03a | 1.12E-03a | 1.12E-03a | multiple sugar transport system permease protein |
| K02027 | 1.31E-03a | | 1.08E-03a | | 1.87E-03a | 1.94E-03a | 1.68E-03a | 1.70E-03a | multiple sugar transport system substrate-binding protein |
| K10543 | 1.14E-04a | | 1.89E-04a | | 1.97E-04a | 1.77E-04a | 1.71E-04a | 1.53E-04a | D-xylose transport system substrate-binding protein |
| K10544 | 1.07E-04a | | 1.57E-04a | | 1.85E-04a | 1.72E-04a | 1.57E-04a | 1.40E-04a | D-xylose transport system permease protein |
| K10545 | 7.02E-05b | | 1.58E-04a | | 1.56E-04a | 1.56E-04a | 1.43E-04ab | 1.23E-04ab | D-xylose transport system ATP-binding protein |
| K10552 | 4.31E-05a | | 6.15E-05a | | 6.64E-05a | 5.41E-05a | 4.97E-05a | 5.34E-05a | fructose transport system substrate-binding protein |
| K10553 | 3.26E-05a | | 4.03E-05a | | 3.95E-05a | 3.73E-05a | 3.21E-05a | 3.24E-05a | fructose transport system permease protein |
| K10554 | 3.88E-05a | | 4.61E-05a | | 4.88E-05a | 4.23E-05a | 4.25E-05a | 3.68E-05a | fructose transport system ATP-binding protein |

**Supplementary Table S8** Soil properties with significant correlation (*P* < 0.05) to microbial community (genus level) and KEGG pathway and CAZy level determined by Monte Carlo permutation tests.

|  | Genus level | | KEGG pathway | | CAZy level | |
| --- | --- | --- | --- | --- | --- | --- |
|  | r | p | r | p | r | p |
| TC | 0.121 | 0.33 | 0.098 | 0.423 | 0.096 | 0.403 |
| TN | 0.455 | **0.004** | 0.689 | **0.001** | 0.612 | **0.001** |
| NH_4_^+^-N | 0.240 | 0.099 | 0.052 | 0.598 | 0.078 | 0.478 |
| NO_3_^-^-N | 0.089 | 0.48 | 0.090 | 0.408 | 0.038 | 0.731 |
| AP | 0.394 | **0.014** | 0.418 | **0.016** | 0.522 | **0.005** |
| AK | 0.208 | 0.146 | 0.380 | **0.021** | 0.142 | 0.268 |
| PH | 0.324 | **0.039** | 0.671 | **0.001** | 0.458 | **0.003** |
| EC | 0.032 | 0.769 | 0.261 | 0.074 | 0.257 | 0.091 |
| SIR | 0.457 | **0.003** | 0.447 | **0.004** | 0.363 | **0.017** |
| MBC | 0.319 | **0.009** | 0.145 | 0.213 | 0.167 | 0.205 |
| MBN | 0.289 | 0.058 | 0.124 | 0.26 | 0.149 | 0.234 |
| Invertase | 0.479 | **0.005** | 0.597 | **0.001** | 0.569 | **0.002** |
| Protease | 0.594 | **0.002** | 0.812 | **0.001** | 0.641 | **0.001** |
| Urease | 0.162 | 0.236 | 0.141 | 0.286 | 0.223 | 0.137 |
| Celluase | 0.125 | 0.309 | 0.322 | **0.033** | 0.269 | 0.087 |
